# Derivation and validation of a prognostic prediction rule from clinical and stress CMR data characterizes cardiac prognostication in patients with suspected myocardial ischemia

**DOI:** 10.1186/1532-429X-13-S1-O4

**Published:** 2011-02-02

**Authors:** Otavio R Coelho-Filho, François-Pierre Mongeon, Michael Jerosch-Herold, Raymond Y Kwong

**Affiliations:** 1 Brigham and Women's, Boston, MA, USA

## Objectives

We sought to derive and validate a parsimonious predictive rule incorporating clinical and stress CMR data to characterize major adverse cardiac events (MACE) in patients with suspected ischemia.

## Methods/results

**Figure 1:**
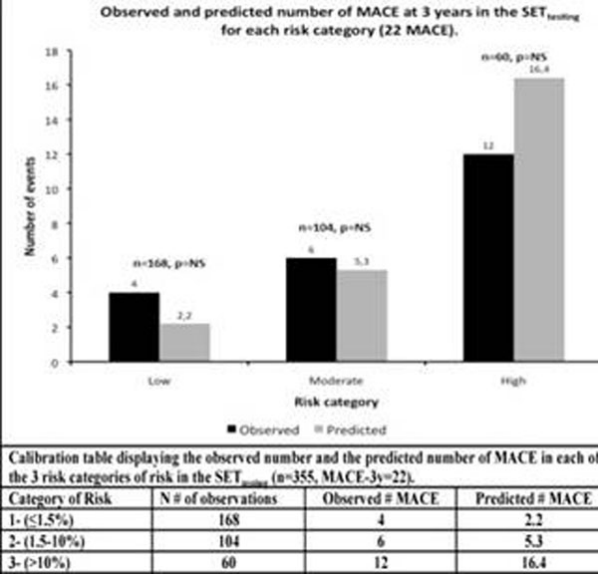
Calibration tables displaying the observed number and the predicted number of MACE in each of the 3 risk categories of risk in the SETtesting (n=365, MACE-3y=22)

Out of 736 patients referred for assessment of suspected ischemia by vasodilator stress perfusion CMR, 25 (3%) were excluded due to images of insufficient quality. The remaining 711 patients (297 females, mean age 56±15 years) were followed for cardiac events (MACE) that occurred within the first 3 years after CMR (100% complete follow-up; median of 21.4 months, range 2.5 months to 8.2 years). We randomized patients in this clinical cohort 1:1 into a training set (SET_Training_, n=356) and a testing set (SET_Testing_, n=355). Forty-six (6.5%) patients experienced MACE: 24 (7%) in the SET_Training_ and 22 (6%) in the SET_Testing_. A parsimonious prediction rule was built by stepwise regression in the SET_Training_, considering all clinical, ECG, and CMR parameters using P<0.05 for both entry and stay. History of PCI, wall motion abnormality at rest, non-sinus rhythm, and CMR myocardial ischemia score (ISCH-SCORE) formed the prediction rule (Table-1). This prediction rule was validated in the SET_Testing_ divided into 3 progressive categories based on their ascending predicted probabilities of MACE (low: 1.5 to 10% and high:>10%). The utility of the model was first determined by comparing the predicted and observed numbers of MACE in each risk category in the SET_Testing_ (Figure-1).

**Table 1:** Best overall Model for MACE (SET_TRAINING_, n=356, MACE at 3 years=23

Variable	Estimate	LR_Χ_^2^	OR	P-Value	Final Model
**ISCH-SCORE, per unit**	0.2104	46.3	1.234	<0.0001	**LR_Χ_^2^ = 49.9**
**Wall Motion Rest**	1.6838	15.7	5.386	<0.0001	**AUC=83%**
**Non sinus rhythm**	1.4101	5.3	4.096	0.02	
**Hx PCI**	0.9691	4.7	2.635	0.04	

**Figure 2:**
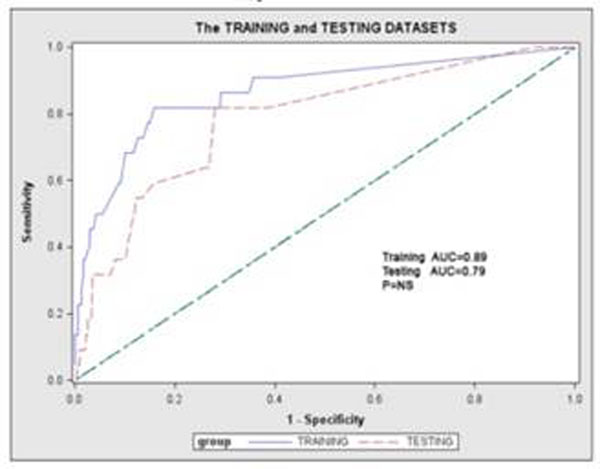
ROC curves of the prediction models derived from the SETt_raining_ (blue line, AUC=86% and applied to SET_testing_ (red line, AUC=79%).

We then performed ROC analyses, in either patient set and both sets combined, to determine how well the range of probabilities by the prediction rule can determine MACE occurence. MACE prediction was excellent in SET_Training_, SET_Testing_, and both sets combined (AUC 83%, 86%, and 79%, P=NS, figure-2).

In addition, we performed an internal validation of the same prediction rule using 400 bootstrap simulations. Each bootstrap sample was the same size as the original derivation sample, but patients were drawn randomly with replacement from the sample. By bootstrap simulation the prediction rule’s estimated optimism was very low (8%±3) and mean AUC of the prediction rule was 82.4% (95%CI: 70%-93%) reinforcing its validity and utility (table-2).

**Table 2:** Bootstrap International Validation for the Prediction Rule: Based on 400 Simulations

	Mean	Minimum	Maximum
Meal Model AUC	82.4 % ± 3.4	70%	93%
Model’s Optimism	7% ± 3.6	-11%	12%

## Conclusion

We derived and validated a prediction rule for MACE using clinical and ISCH-SCORE for patients with suspected myocardial ischemia. ISCH-SCORE provided strong and major prognostic utility in this prediction rule.

